# Antimicrobial Activity of Azithromycin Encapsulated into PLGA NPs: A Potential Strategy to Overcome Efflux Resistance

**DOI:** 10.3390/antibiotics11111623

**Published:** 2022-11-14

**Authors:** Yasmin Abo-zeid, Amr Amer, Marwa Reda Bakkar, Boushra El-Houssieny, Wedad Sakran

**Affiliations:** 1 Department of Pharmaceutics and Industrial Pharmacy, Faculty of Pharmacy, Helwan University, Cairo 11795, Egypt; 2 Helwan Nanotechnology Center, Helwan University, Cairo 11792, Egypt; 3 National Organization for Drug Control and Research (NODCAR), Giza 12511, Egypt; 4 Botany and Microbiology Department, Faculty of Science, Helwan University, Cairo 11795, Egypt

**Keywords:** azithromycin, PLGA nanoparticles, antimicrobial activity, resistant bacteria, efflux resistance mechanism

## Abstract

Antimicrobial resistance represents a public health problem with a major negative impact on health and socioeconomic development, and is one of the biggest threats in the modern era. This requires the discovery of new approaches to control microbial infections. Nanomedicine could be one of the promising strategies to improve the treatment of microbial infections. Polymer nanoparticles (PNPs) were reported to overcome the efflux-resistant mechanism toward chemotherapeutic agents. However, to the best of our knowledge, no studies were performed to explore their ability to overcome the efflux-resistant mechanism in bacteria. In the current study, azithromycin (AZI), a macrolide antibiotic, was encapsulated into a biocompatible polymer, poly (lactic-co-glycolic acid) (PLGA) using the nano-precipitation method. The effect of the drug to polymer ratio, surfactant, and pH of the aqueous medium on particle size and drug loading percentage (DL%) were investigated in order to maximize the DL% and control the size of NPs to be around 100 nm. The antibacterial activity of AZI-PLGA NPs was investigated against AZI-resistant bacteria; Methicillin-resistant *Staphylococcus aureus* (MRSA) and *Enterococcus faecalis* (*E. faecalis*), where the efflux mechanism was demonstrated to be one of the resistant mechanisms. AZI-PLGA NPs were safer than free AZI, as revealed from the cytotoxicity test, and were able to overcome the efflux-resistant mechanism, as revealed by decreasing the MIC of AZI-PLGA NPs by four times than free AZI. The MIC value reduced from 256 to 64 µg/mL and from >1000 to 256 µg/mL for MRSA and *E. faecalis*, respectively. Therefore, encapsulation of AZI into PNPs was shown to be a promising strategy to overcome the efflux-resistant mechanism towards AZI and improve its antibacterial effect. However, future investigations are necessary to explore the effect (if any) of particle size, surface charge, and material composition of PNPs on antibacterial activity. Moreover, it is essential to ascertain the safety profiles of these PNPs, the possibility of their large-scale manufacture, and if this concept could be extended to other antibiotics.

## 1. Introduction

Antimicrobial resistance (AMR) occurs when microorganisms, including bacteria, viruses, fungi, and parasites, become able to adapt and grow in the presence of antimicrobial agents that once impacted them [1,2]. Globally, the estimated number of deaths due to resistant microorganisms is around 700,000 per year [3,4]. Antibiotics currently available in the market are able to attack bacteria at a single site [5,6,7]. This increased the possibility of pathogenic microorganisms to develop resistance against antibiotics rendering them inactive [8,9].

In 2017, a list of antibiotic-resistant pathogens, including both Gram-positive and Gram-negative bacteria was reported [10]. Although there are many mechanisms developed by bacteria to resist antibiotics [11], all of these bacteria reported antibiotic resistance mediated by efflux pumps [10]. This highlighted the impact and importance of efflux pumps in the clinical setting [12]. Antibiotic resistance, mediated by efflux, involves bacterial expression of efflux pumps, such as the ATP-binding cassette transporters or the major facilitator superfamily transporters that transport tetracyclines, glycopeptides, and macrolides out of cells to efflux antibiotics outside the bacterial cell and consequently increasing the minimum inhibitory concentration of antibiotics, or in some cases rendering them completely ineffective [13]. Administration of high doses of antibiotics to attain the effective concentration of antibiotics required to kill pathogens might be associated with adverse effects such as alteration of the growth of harmless and useful organisms (e.g., bacteria in the intestine) [14,15] and/or toxic effects may be developed in some cases due to accumulation of antibiotics at off-target sites [14,15]. For example, prolonged administration of high doses of the aminoglycoside, tobramycin, can cause acute and chronic nephrotoxicity by decreasing glomerular filtration rate and altering excretion of electrolytes [16,17].

Therefore, finding a selective delivery system that could deliver antibiotics to the infected sites while decreasing or avoiding their accumulation at off-target tissues and being able to overcome efflux-mediated resistance mechanisms could result in a dramatic improvement of the antibacterial activity of antibiotics. Nanoparticles (NPs) are an advanced delivery system, and interest in their clinical development for different diseases, including combating infections, has been growing over the last few years [4,7,8,18,19,20,21,22,23,24,25,26,27,28,29,30,31]. This is mainly attributed to their unique physicochemical properties, such as small particle size ranging from 1 to 1000 nm and a high surface area [32], being able to control/sustain drug release [33,34], deliver the antibiotics selectively at high concentrations into the infected sites, and thus, there is less possibility of bacteria developing resistance [15,35,36]; moreover, a lower possibility of drug accumulation at off-target sites [14,37]. In addition, NPs are also protecting the drug against degradation [38], and they are taken up by bacteria through a different route than free antibiotics [39,40].

Only a small number of studies have reported the application of nanotechnology to overcome bacterial efflux resistance, for example, metal-based nanoparticles (MBNPs) were able to overcome drug efflux mechanisms [41,42,43,44] through direct binding to the active site of efflux pumps, thereby blocked the extrusion of antibiotics outside the cells or they might disrupt efflux kinetics [44]. However, MBNPs are still required to undergo further investigations to address their biosafety profiles and ascertain their clinical application [7,27]. Moreover, several reports on bacterial resistance to MBNPs are gradually emerging [28,45].

In recent work performed by Raza and Colleagues [46], they reported the capability of polyethylene glycol (PEG) grafted porous silicon nanoparticles (a biocompatible and biodegradable nanomaterial) to overcome the efflux system responsible for ultra-low oral bioavailability of meropenem (a carbapenem antibiotic), and thus, improving the antibacterial activity of meropenem and increasing the potential of its oral administration in the future. Other studies reported the encapsulation of antibiotics into solid lipid nanoparticles to overcome the drug efflux activity in yeast [47] and mycobacteria [48].

Encapsulation of antibiotics into polymer nanoparticles (PNPs) were reported to improve their antibacterial activity [28,29,49]. However, according to the author’s knowledge, the application of PNPs as a delivery system for antibiotics to overcome the bacterial efflux-resistant mechanism has not been studied yet. PNPs loaded with chemotherapeutic agents was reported to bypass the efflux transporter, and this was attributed to different cellular entry mechanism of NPs compared to the free drug where PNPs are taken up into cancer cells through endocytosis instead of the diffusion and release of the drug at a perinuclear site within the cell, away from cell membranes and efflux pumps [50,51]. Therefore, we hypothesized that encapsulation of antibiotics into PNPs might act similarly with bacteria and be able to overcome bacterial efflux resistance.

To ascertain our hypothesis, we encapsulated azithromycin (AZI), a macrolide antibiotic into Poly (lactic-co-glycolic acid) polymer (PLGA) NPs using the nano-precipitation method, the effect of the drug to polymer ratio, surfactant, and pH of the aqueous medium on the particle size and drug loading percentage (DL%) were investigated. This was followed by investigating the cytotoxicity of the free AZI versus selected nano-formulation on Wi-38 cells, human lung fibroblast cell lines. The antibacterial activity of selected formulations of AZI-PLGA NPs was explored against bacterial strains previously demonstrated to carry efflux mechanisms and were resistant to AZI; methicillin-resistant *Staphylococcus aureus* (MRSA), and *Enterococcus faecalis* (*E. faecalis*). In addition, Gram-negative bacteria *Pseudomonas aeruginosa*, that is lacking an efflux mechanism, was also tested.

Azithromycin, was used in the current study because it is a relatively inexpensive antibiotic and is often deemed a wonder drug due to its safety and effectiveness against parasitic and helminth infections, in addition to a wide range of bacterial infections [52]. Moreover, it was one of the medicines proposed as a potential therapy for the treatment of SARS-CoV2 pneumonia due to its antimicrobial and immunomodulatory activity [53,54]. Furthermore, biannual administration of azithromycin (mass drug administration, MDA) was recommended for trachoma control, and it was found to reduce all-cause mortality [55]. However, this was reported to amplify bacterial resistance toward AZI [56]. The literature reported bacterial efflux resistance mechanism against macrolides in both Gram-positive and Gram-negative bacteria [57,58,59]. PLGA was used in the current work due to its degradability and compatibility with the biological system [60,61].

## 2. Materials and Methods

### 2.1. Materials

AZI and PLGA (50:50) acid terminated with a molecular weight of 17 KDa were a kind gift from Pharco Alexandria, Egypt and Corbion, Netherlands, respectively. HPLC column (C_18_, 250 mm × 4.6 mm, 5 µm) was obtained from ACE, Egypt. Dipotassium dihydrogen phosphate, potassium dihydrogen phosphate, sodium hydroxide, and dimethyl sulfoxide (DMSO) were supplied by Merck, Germany. HPLC grade water was obtained from Milli-Q direct water purification system, Merck Millipore, USA. HPLC-grade organic solvents, ethidium bromide (EtBr), 3-(4,5-dimethylthiazol-2-yl)-2,5-diphenyl tetrazolium bromide (MTT), and buffered peptone saline (BPS) tablets pH 7.2 were purchased from Sigma-Aldrich, Germany. EtBr stock solution was prepared in double distilled water at a concentration of 50 mg/mL, and stored protected from light at 4 °C to be further diluted upon performing the experimental work. Verapamil was provided from Pharaonia Pharmaceuticals, Cairo, Egypt. Verapamil solution was prepared in desired concentrations in BPS, pH 7.2. All microbiological media used in this study were dehydrated and freshly prepared for each experiment and were prepared according to the manufacturer’s instructions. Muller–Hinton broth (MHB) was purchased from HiMedia (HiMedia Laboratories Pvt. Ltd., Mumbai, India), tryptone soy broth (TSB) and agar (TSA) were purchased from Biolife (Biolife Italiana srl, Milano, Italy).

Three pathogenic bacterial strains were used in this study; AZI resistant strain, *Enterococcus faecalis* that was kindly provided by Prof., Dr Mahmoud Yassein and Mr Akram Nader, Microbiology and Immunology Department, Faculty of Pharmacy, Ain Shams University, Cairo, Egypt [62]. *Pseudomonas aeruginosa* and methicillin-resistant *Staphylococcus aureus* (MRSA) were obtained from the culture collection of the Bacteriology laboratory, Botany and Microbiology Department, Faculty of Science, Helwan University. Both strains were demonstrated to have efflux mechanisms to resist AZI.

### 2.2. Methods

#### 2.2.1. Preparation of AZI-PLGA NPs

AZI-PLGA NPs were prepared by nanoprecipitation method following a previous protocol [63], with the following modifications; PLGA (50 mg) and AZI (10, 15, 20, or 50 mg) were dissolved in acetone (2 mL) to form the organic phase. The organic phase was added dropwise (1 mL/min) into the aqueous phase (15 mL) under sonication using a probe sonicator (UP50H, Hielscher ultrasound technology, Teltow, Germany). The samples were left to stir overnight at room temperature for complete evaporation of the organic solvent. Several aqueous phases were used; water, water in the presence of Tween 80 (0.1% *v*/*v*), and phosphate buffer 10 mM, pH 6, 7.4, and 8. Then after, NPs were separated by centrifugation (Hermle Z446-K Refrigerated Centrifuge) at 22,095 RCF for 1 h at 12 °C. The supernatant was discarded and NPs pellets were washed by the corresponding aqueous phase (15 mL × 5). The washed NPs were dried under vacuum at 25 °C (Memmert VO200, Schwabach, Germany) until constant weight. Dried NPs were stored in an airtight desiccator for further experimental work. Each sample was prepared as a triplicate.

#### 2.2.2. Characterization of Nanoparticles

##### Particle Size and Zeta-Potential

The dried NPs were re-dispersed in the corresponding aqueous phase by probe sonication for 30 sec (UP50H, Hielscher ultrasound technology, Germany). The particle size and zeta potential were measured using Malvern Zeta sizer Nano ZS (Malvern Instruments Ltd., Malvern, UK). Samples were diluted with the corresponding aqueous phase to give a count rate of 50 to 300 Kcps, and measurements were performed at 25 °C ± 0.1.

##### Drug Loading

AZI drug loading was determined using a previously reported protocol [64]. A known weight of the dried NPs was dissolved in acetonitrile (1 mL), then methanol (3.5 mL) and phosphate buffer (10 mM, pH 8, 0.5 mL) were added and this was followed by centrifugation at 22,095 RCF for 1 h, at 12 °C. The obtained supernatant (200 µL) was analyzed by injection into the HPLC column, ACE C_18_ column (250 mm × 4.6 mm, 5 µm). Methanol:phosphate buffer (10 mM, PH 8) in a ratio of 90:10 was used as a mobile phase, the elution was isocratic at a flow rate of 1.5 mL/min, temperature 50 °C, and UV detection was performed at 210 nm. The total amount of AZI was calculated from a calibration curve of different known AZI concentrations (5 to 320 μg/mL) dissolved in acetonitrile: methanol: phosphate buffer (10 mM, pH8) at a molar ratio of 1:3.5:0.5, respectively and analyzed under the previously described condition. The percentage of drug loading was calculated using Equation (1)

(1) $$\mathrm{Drug}\;\mathrm{loading}\;\%=\frac{Amount\;of\;encapsulated\;drug\;\left(mg\right)}{Weight\;of\;nanoparticle\;\left(mg\right)\;}*100$$

To identify the efficiency of AZI extraction from PLGA NPs, we followed our previously published protocol [26] with the following modifications; known weights of PLGA (10 mg) and AZI (30, 70, 100 µg) were dissolved in acetonitrile (1 mL) to dissolve all components followed by addition of methanol (3.5 mL) and the phosphate buffer (10 mM, pH8, 0.5 mL) to precipitate PLGA. Then, all samples were centrifuged before injection into the HPLC column to be analyzed as described earlier. The efficiency of the extraction was determined via the calculation of the recovery percentage as presented in Equation (2);

(2) $$\mathrm{Recovery}\;\%=\frac{Peak\;Area\;ofAZI\;\left(sample\right)}{Peak\;area\;of\;AZI\;\left(refernce\right)}\;*\;100$$

The peak area of AZI (sample) was calculated for the known AZI concentrations that had been extracted from the AZI samples. The peak area of AZI (reference) was calculated for pure AZI samples of similar concentrations that had been injected directly into HPLC without any pre-treatment.

##### Validation of HPLC Analysis

The HPLC analysis of AZI was validated by determination of linearity, inter, and intra-day variation following our previous protocol [26], with the following modifications; the linearity of AZI was determined for AZI concentrations ranging from 5 µg to 320 µg dissolved in acetonitrile:methanol:phosphate buffer (10 mM, pH8) at a molar ratio of 1:3.5:0.5, respectively. Then afterward, the samples were injected into the HPLC column to be analyzed under the conditions previously described in Section 2.2.2.

The accuracy and precision of inter-day and intra-day variation were determined by using AZI concentrations of 30, 70, and 100 μg/mL. The analysis of these concentrations was performed three times on the same day at different times (intra-day variation) or on different days (inter-day variations). The accuracy and precision were calculated using Equations (3) and (4), respectively.

(3) $$\mathrm{Accuracy}=\frac{\left(M-N\right)}{\left(N\right)}*100$$

M is the mean value of AZI concentration measurements, while N is the theoretical concentration.

(4) $$\mathrm{Precision}\;(\%\;\mathrm{RSD})=\frac{SD}{M}*100$$

RSD is the relative standard deviation; SD is the standard deviation of measurements, and M is the mean value of AZI measurements.

##### Transmission Electron Microscopy (TEM)

Selected formulations of AZI-PLGA NPs were imaged using TEM (H-700, Hitachi Ltd., Japan) at an accelerated voltage of 80 kv using a negative staining method following our previously published protocol [30]. Samples were diluted (1:50) with double distilled water, then, a drop of diluted sample was applied on a mesh copper grid coated with carbon film and was kept for 5 min to dry, then after, a drop of phospho–tungstic acid (2% *w*/*w*) was added on the grid for 50 sec and the excess liquid was removed using filter paper. The grid was left to air-dry in advance of imaging. TEM imaging was performed for the identification of the sample uniformity, presence of aggregation (if any), shape, and size.

##### Differential Scanning Calorimetry (DSC)

Thermograms of pure powder of AZI, PLGA, selected formulations of AZI-PLGA NPs and physical mixture (PM) of AZI and the polymer were recorded on a DSC (Perkin Elmer, Waltham, MA, USA) following the previously published protocol [63] with the following modifications; samples (5 mg) were placed in aluminum pans and covered by aluminum lids. The thermal behavior of the samples was recorded at a scanning rate of 10 °C/min over a temperature range of 25 to 200 °C to cover the melting point of PLGA and AZI; 50 °C and 126 °C, respectively. The instrument was calibrated using the indium standard.

##### Release Study

Selected formulations of AZI-PLGA NPs, pure AZI solution, and AZI suspension were studied for drug release following the previous protocol [65] with the following modifications; the known weight of the sample containing 2.33 mg of AZI was dispersed in 2 mL of the release media (PBS, 10 mM, pH 7.4 containing 10% *v*/*v* ethanol). The dispersed samples were placed into a dialysis membrane bag (dimensions are 7 cm × 2.5 cm, pore size 2.4 nm, molecular weight cutoff 12–14 KDa) that was previously equilibrated with the release medium for 24 h. The release study was initiated by placing the dialysis bags containing samples in the release medium (20 mL) at 37 °C and under stirring (50 rpm). Samples (3 mL) were withdrawn and replaced with an equal volume of fresh release medium at pre-determined time points. The experiment was run for 17 days and each time-point was run in triplicate and analyzed by HPLC as was previously explained in Section 2.2.2. AZI solution was prepared in ethanol, and AZI suspension was dispersed in the corresponding aqueous medium.

The mechanism of AZI release from AZI-PLGA NPs was determined by using DD Solver software, as previously reported [66]. The release data were fitted for several release kinetic models. The mathematical model that best fit the kinetic release profile was selected based on the highest coefficient value of R^2^.

#### 2.2.3. Cytotoxicity Assay

**Cell Culture**

Wi-38 cells, human lung fibroblast cell lines were cultured in a complete DMEM culture medium containing FBS (10% *v*/*v*), Earle’s balanced salt solution, non-essential amino acids, L-glutamine (2 mM), sodium pyruvate (1 mM), sodium bicarbonate (1500 mg/L), penicillin G sodium (10.000 UI), streptomycin (10 mg), and penicillin (25 µg), followed by incubation of cells at 37 °C and 5% CO_2_, and the culture medium was refreshed every 24 h. As the density of cells was 70 to 90%, they were sub-cultured to achieve the desired density for the cytotoxicity test.

For the sub-culture, the cell culture medium was first removed, and the flask was washed twice with PBS. To detach the cells from the culture flask, trypsin/EDTA was deposited; subsequently, this cell suspension was mixed with a fresh complete culture medium in Falcon tubes. Finally, the cells were collected by centrifuging at 1500 rpm for 5 min and then adjusted to the destiny required for the cytotoxicity test.

**Neutral Red Cytotoxicity Assay**

Wi-38 cells (1.5 × 10^4^ cells/well) were seeded into 96-well culture plates. Complete culture medium (100 µL; DMEM with FBS (10% *v*/*v*), non-essential amino acids, L-glutamine (2 mM), sodium pyruvate (1 mM), sodium bicarbonate (1500 mg/L), penicillin G sodium (10.000 UI), streptomycin (10 mg) and amphotericin B (25 µg) was added to the cells, followed by incubation for 24 h at 37 °C and 5% CO_2_. Tested samples were diluted with a complete culture medium to prepare a set of concentrations ranging from 1000 to 31.25 ug/mL for F5, F10, and their blank samples (NPs prepared similarly but with the absence of AZI). Subsequently, the complete culture medium was removed from each well, and the diluted tested samples (200 µL) were added, followed by incubation for 24 h at 37 °C and 5% CO_2_. Then, the culture medium containing the formulations was removed and the cells were washed with PBS (150 µL × 2). Then, after the neutral red solution (100 µL, 40 µg/mL) was added and the cells were incubated under the same condition for 2 h. Then, a neutral red solution was aspirated, followed by a cell wash with PBS (150 µL × 2), then the addition of de-stain solution (10 mL of Ethanol 96%, 10 mL of deionized water, and 0.2 mL of glacial acetic acid).

Finally, a microplate reader (Multilabel Plate Reader, PerkinElmer, Boston, MA, USA) was used to measure the absorbance of the treated cell suspension at a wavelength of 490 nm. The percentage of cell viability was calculated by dividing the test absorbance by the control absorbance and multiplying the result by 100.

#### 2.2.4. Antibacterial Study

##### Azithromycin Susceptibility Test

The susceptibility of MRSA and *Pseudomonas aeruginosa* towards free AZI was evaluated by the disc diffusion method following the standard of the Clinical and Laboratory Institute [67] where standard AZI discs (containing 15 µg of free AZI) were used. AZI-resistant bacterial strain (*Enterococcus faecalis*), due to the efflux mechanism, was used as a positive control [62].

##### Determination of Minimum Inhibitory Concentration (MIC)

MIC (the lowest concentration of the antibiotic inhibited visible growth of bacteria) of free AZI was determined using a microdilution assay [67]. Briefly, bacterial isolates, *Pseudomonas aeruginosa*, MRSA and *Enterococcus faecalis* were grown overnight in TSB at 37 °C until an OD_600_ of 0.8 was reached. Two-fold serial dilutions of AZI in MHB were prepared with a concentration range of 0.125 to 512 µg/mL, and 100 µL of each concentration was inoculated into 96 wells plate followed by the addition of 5 µL of the final inoculum of bacteria containing 5 log CFU/mL. Inoculated plates were then incubated for 18–24 h at 37 °C (Stuart S150 Orbital Incubator). Then, MTT reagent (10 μL, PBS 7.2, 5 mg/mL) was added to each well and the plates were incubated again at 37 °C for 4 h. Then after, the wells were aspirated and DMSO (200 μL) was added, followed by plate incubation at 37 °C for another 2 h to solubilize the formed purple crystals of formazan. The absorption of samples was measured at 570 nm using an acculab MR-90 microplate reader. A set of control wells was prepared; (1) positive control: broth medium inoculated with tested bacteria (5 µL, 5 log CFU/mL), (2) negative control wells: sterile broth medium only with the absence of free AZI and tested bacteria; (3) sterile broth medium with free AZI solution. Each sample/control was prepared in triplicate.

##### Investigation of Bacterial Efflux Activity

- Determination of efflux activity by cartwheel method

The efflux activity of the tested bacterial strains was investigated using EtBr agar cartwheel method following a previously reported protocol [68]. Briefly, TSA agar plates containing EtBr concentrations ranging from 0 to 2.5 µg/mL were prepared on the same day of the experiment and protected from light. Then, fresh bacterial cultures of 5 log CFU/mL were prepared and swabbed over the surface of the prepared agar starting from the center toward the edge of the plate, followed by overnight incubation at 37 °C. Incubated plates were then examined under a suitable source of UV light (Long-Wave UV Pen Light 1660530EDU, Biorad, Hercules, CA, USA.).

- Determination of AZI efflux using efflux pump inhibitor (EPI)

Verapamil (VP), an calcium channel blocker drug commonly used for the treatment of hypertension [69], was previously reported to act as EPI for both Gram-negative and Gram-positive bacteria [70,71]. The effect of VP as an EPI on the MIC value of AZI for MRSA, *Pseudomonas aeruginosa* and *Enterococcus faecalis* was explored. Initially, the MIC value of verapamil was initially determined by microdilution assay [67] as described previously in Section 2.2.4, where VP was prepared in PBS pH 7.4 with a concentration range from 0.125 to 2 mg/mL. Then after, the effect of VP on the MIC value of free AZI was determined using a constant concentration of VP; ½ × MIC. Consequently, wells were inoculated with two-fold serial dilutions of free AZI ranging from 0.125 to 512 µg/mL, followed by ½ × MIC of VP. Then after, wells were inoculated with bacterial inoculum of 5 log CFU/mL. The plates were incubated at 37 °C and examined for the lowest free AZI concentration inhibited the visible growth of bacteria. The presence of a specific AZI efflux mechanism was confirmed when the MIC value of free AZI decreased by more than 4-fold in the presence of VP [72].

- Determination of MIC for AZI-PLGA NPs

The MIC values for selected formulations of AZI-PLGA NPs (F5 and F10) were determined using microdilution assay [67], as described previously. In addition to the previous control sets stated in Section 2.2.4, other control sets were prepared; wells contained growth medium and AZI-PLGA NPs (F5/F10), and other wells contained growth medium inoculated with bacteria and Blank PLGA NPs. Each sample was prepared in triplicate.

#### 2.2.5. Statistical Analysis

All statistical analysis was performed using one-way and two-way ANOVA. Analyses were carried out by GraphPad Prism 9.0 software at a confidence level of 95%.

## 3. Results and Discussion

### 3.1. Characterization of AZI-PLGA NPs

#### 3.1.1. Particle size and Zeta Potential

The particle size was presented as an average value (Dnm) ± standard deviation (SD), Table 1. The size obtained for most AZI-PLGA NPs was ranging from 100 to 225 nm. Increasing the initial amount of AZI, the presence of Tween 80, or sitting pH of the aqueous medium at 6, 7.4, and 8 reduced the particle size non-significantly (*p* > 0.05) compared to the Blank PLGA NPs except for samples prepared at AZI (50 mg). The later samples showed a significantly (*p* < 0.05) bigger particle size (size > 400 nm) and a non-significant (*p* > 0.05) smaller particle size in the presence of Tween 80 compared to Blank PLGA NPs.

Most of AZI-PLGA NPs were monodispersed, as indicated by PDI values (PDI ≤ 0.3) except NPs prepared at AZI (50 mg) and pH 8 (PDI > 0.3) indicating polydisperse samples (Table 1). This is consistent with our previous study [30], and other studies reported [73,74,75] that PDI < 0.3 is indicative of good homogeneity and is suitable for drug delivery applications.

PDI values > 0.3 might be explained by the lower solubility of AZI and its tendency to precipitate at high concentration, moreover, deionization of AZI at alkaline pH enhanced the drug precipitation and destabilization of the system [76]. Although AZI-PLGA NPs were prepared in the current study using a modified method to what was reported by Mohammadi and colleagues [63], the obtained particle size was close to the size (212 to 252 nm) reported by Mohammadi and colleagues.

The stabilization effect of Tween 80 in preventing particles from aggregation is more pronounced for NPs prepared at an initial weight of AZI, 50 mg. As presented in Table 1, the PDI value (0.27) recorded for NPs prepared in the presence of Tween 80 were significantly (*p* < 0.05) lower than PDI values (0.437 to 0.651) recorded for samples prepared in the absence of Tween 80. This might be explained by the stabilizing steric effect of Tween 80, being able to interact with the surface of NPs and forming a single monolayer around NPs, and this resulted in a lower possibility of particle aggregation [77,78]. Tween 80 is a non-ionic surfactant, able to reduce the surface tension of the hydrophobic polymer when it comes into contact with the aqueous phase and thus promoting the polymer assembly to form NPs [79]. The effect of Tween 80 obtained in the current study is consistent with our previous study, where Tween 80 decreased both particle size and the tendency of particle aggregation for NPs prepared with acylated poly(glycerol-adipate) polymer [26]. Another study performed by Navneet and colleagues demonstrated a similar stabilizing effect of Tween 80 (0.05%), as it reduced the tendency of paclitaxel PLGA NPs aggregation, in addition, reduced their particle size from 438 to 389 nm [80].

The zeta potential data for all NPs were presented as zeta potential (mv) ± SD, where all sample measurements were performed in the corresponding aqueous phase, as described in Section 2.2.2. All NPs were stable, as indicated by the high negative zeta potential value, ranging from −30 to −52 mv (Table 1). The negative surface charge is attributed to the free COOH group in each polymer chain that could result in charge stabilization and, therefore, a lower possibility of aggregate formation. As revealed in Table 1, Tween 80 had a non-significant (*p* > 0.05) effect on the zeta potential value, and this might be due to the lower concentration (0.1% *v*/*v*) used in the current study. Similarly, pH has a non-significant (*p* > 0.05) effect on the zeta potential value, where pH ≥ 6 might be considered alkaline enough to ionize COOH groups in polymer chains. Our results are consistent with a previous study [26], where Tween 80 did not affect the zeta potential of nanoparticles prepared from different derivatives of poly (glycerol-adipate) polymer, and it was explained by low coverage of particle surface by Tween 80 due to the short chain length of PEG in Tween 80, the low concentration of Tween 80 used and consequently a flattened PEG layer might be formed which was too thin to influence the zeta potential. However, the zeta potential obtained in the current study is much better than the zeta potential values (−5.6 to −15.56 mv) reported by Mohammadi and colleagues [63]; this might be attributed to different drug:polymer ratios, surfactant type/concentration, and methodology applied.

#### 3.1.2. Drug Loading (DL%)

For accurate determination of DL%, the separation of unentrapped AZI from AZI-loaded PLGA NPs was a crucial factor in avoiding biased values of DL%. AZI-PLGA NPs were purified by washing the obtained NPs pellets with a volume of the washing solution that is sufficient to dissolve the initial amount of AZI used for the preparation of NPs to ensure efficient removal of all unentrapped AZI. The complete removal of the unentrapped AZI was further confirmed by the disappearance of its peak after injecting the washing solution into the HPLC column.

Another challenge for the accurate determination of DL% was the efficacy of AZI extraction from AZI-PLGA NPs. The extraction methodology of AZI was described in Section 2.2.2, and it was expressed as the recovery percentage ± (SD). The recovery percentage was found to be 85 ± 5%.

Furthermore, the validation of HPLC analysis of AZI was also performed, as described in Section 2.2.2. The linearity of AZI was demonstrated over a concentration range from 5 to 320 µg/mL, and the correlation coefficient (R^2^) value was 0.9978. The values of the limit of detection (LOD) and limit of quantification (LOQ) were 9.66 and 29.29 μg/mL, respectively. The intra-day and inter-day variations were performed using three different concentrations (30, 70 and 100 μg/mL). The accuracy for intra-day and inter-day variations ranged from 1.42 to 2.05 and from 0.42 to 2.71, respectively. The precision (%RSD) was also calculated and it ranged from 0.36% to 1.88% and from 1.83% to 2% for intra-day and inter-day variations, respectively. The values of precision indicated a high degree of repeatability and reproducibility. In accordance with AOAC guidelines [81], the following equations; % RSD of repeatability = C ^−0.15^, and % RSD of repeatability = 2 C ^−0.15^ were used to calculate the theoretical RSD% for intra-day and inter-day variations, respectively, where C is the analyte concentration expressed as mass fraction. The accepted practical %RSD for a valid method should range between ½ and 2 times the theoretically calculated values. The obtained values matched with what was recommended by AOAC guidelines, and this assures the validity of the HPLC analysis method used in the current study.

In the present work, we are concerned with maximizing the loading percentage of AZI into PLGA NPs to enhance its antibacterial activity against AZI-resistant bacteria. Thus, different factors such as initial AZI amount, presence of surfactant, e.g., Tween 80 (0.1% *v*/*v*) and the pH of aqueous phase were investigated to attain the highest DL%. However, as revealed from Table 1, these factors had a non-significant (*p* > 0.05) effect on DL%, and the obtained DL% ranged from 2.88 ± 0.34 to 5.74 ± 0.28%. Thus, we selected two formulations; F5 (5.74 ± 0.28%) and F10 (4.78 ± 0.13%), for further characterization and microbiological study for the following reasons; (1) they have the highest DL% among all formulations, (2) they might have different surface properties due to adsorption of few molecules of Tween 80 at the surface of F5 formulation and consequently, this might affect their antibacterial activity on tested bacterial strains.

#### 3.1.3. Transmission Electron Microscopy (TEM)

**Figure 1:**
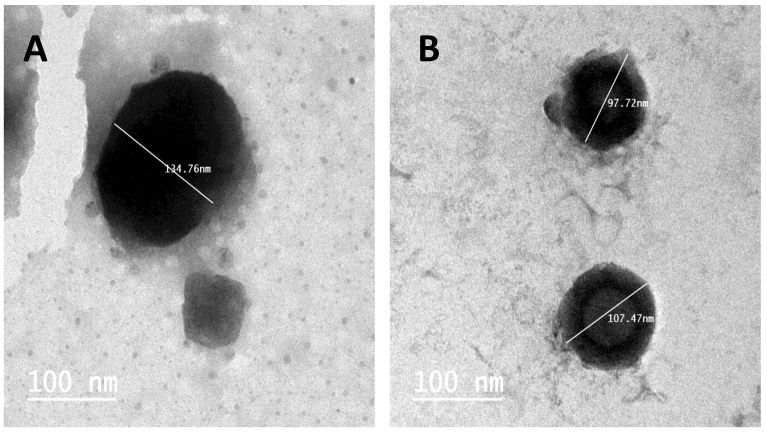
TEM images of AZI PLGA NPs formulations; (**A**) F5 and (**B**) F10, scale bar 100 nm.

TEM was performed for selected AZI-PLGA NPs formulations, F5 and F10. As presented in Figure 1, the particle size of F5 (134 nm) and F10 (97.72 to 107 nm) was close to data obtained by the Malvern instrument. TEM images showed spherical nano-capsules with no sign of particle aggregation.

#### 3.1.4. Differential Scanning Calorimetry

**Figure 2:**
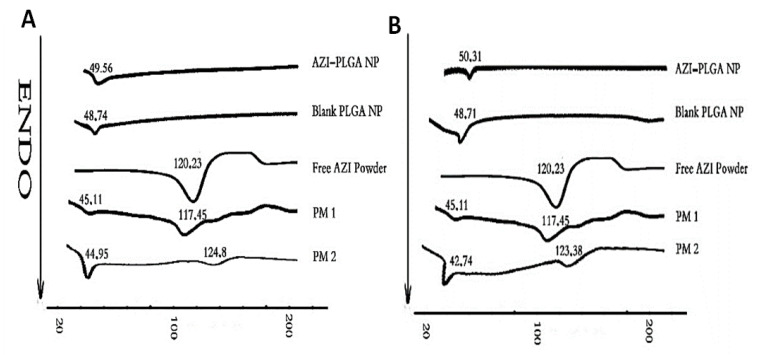
DSC curves for (**A**) F5 and (**B**) F10 formulations in the presence of DSC curves of Blank PLGA NPs, Pure azithromycin powder (AZI), physical mixtures (PM1, AZI:PLGA ratio, 1:1), PM2 (AZI:PLGA ratio similar to the ratio in AZI-PLGA NPs formulation).

DSC scan performed for F5 and F10 formulations, Blank PLGA NPs, AZI pure powder, physical mixtures PM1 and PM2 at a molar ratio of PLGA:AZI equivalent to 1:1 the ratio in AZI-PLGA NPs formulation respectively was presented in Figure 2. The endothermic peak detected for Blank PLGA NPs, and AZI-PLGA NPs was close to each other and was slightly higher than the endothermic peak detected for PLGA in PM1 and PM2, indicating the assembly of PLGA to form NPs. Pure AZI powder had an endothermic peak at 120.23 °C corresponding to its melting point that is very close to its endothermic peak detected for PM1 and PM2 (117.45, 124.8, and 123.38 °C). PM1 has an endothermic peak for AZI with a higher intensity than PM2 due to a higher molar ratio of AZI to PLGA in PM1 compared to PM2. For F5 and F10 NPs formulations, the endothermic peak of free AZI disappeared from the thermograms, which is indicative for encapsulation of AZI into PLGA NPs and the presence of AZI in the amorphous state where it is molecularly dispersed in the polymeric structure of NPs. Our data is consistent with the literature [63], however, the endothermic peak of AZI detected in the current work was recorded at a lower temperature than the reported temperature, 149.33 °C. This might be attributed to different equipment applied for DSC analysis.

#### 3.1.5. Release Study

**Figure 3:**
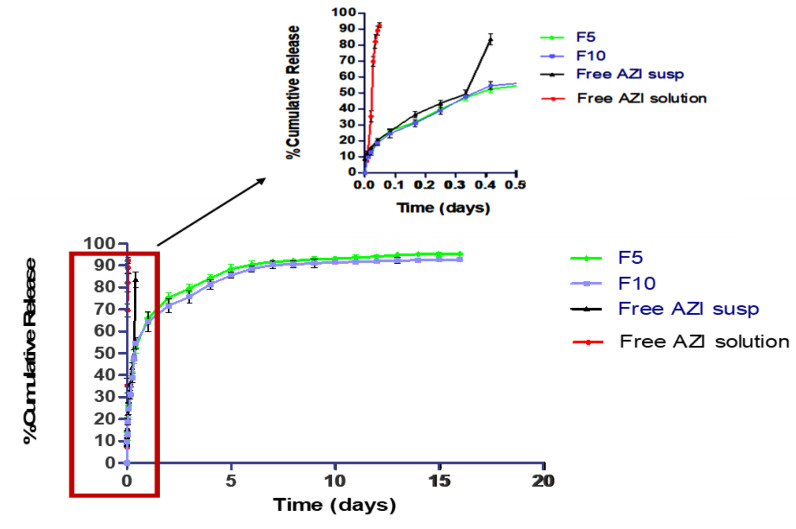
In vitro release of free AZI solution, suspension, F5, and F10 formulations of AZI-PLGA NPs.

Release studies were performed and presented in Figure 3 for the solution, the suspension of free AZI, F5, and F10 formulations. AZI in solution form was released faster than other formulations, where 80% of AZI was released after 50 min. Contrary to this, 10 to 15% of AZI released from suspension and NPs formulations after 1 h; this was followed by a gradual release up to 85% after 1 and 15 days, respectively. As presented in Figure 3, the release of AZI from AZI-PLGA NPs followed a typical biphasic release pattern, where a first burst release (10–15% of free AZI) might be attributed to the large surface area of AZI-PLGA NPs that allowed the loosely bound/adsorbed free AZI at the surface of NPs to diffuse rapidly. Then, the second phase sustained AZI release for 15 days and this might be attributed to the diffusion of encapsulated AZI along the polymer chain matrix [82,83]. Thus, drug encapsulation into polymer NPs sustained the release of the drug to a longer extent than free AZI suspension. Our data is consistent with data reported by Mohammadi and colleagues [63], where incorporation of AZI into PLGA NPs sustained its release but to a lower extent (24 h). In addition, our data is matched with other studies, where paclitaxel incorporation into PLGA nano-formulation sustained the release of paclitaxel for more than 30 days [84].

Release kinetic models were applied to assess the mechanism of AZI release from F5 and F10 NPs formulations. R^2^ values were obtained by linear regression analysis and they were best fitting Higuchi kinetics, where R^2^ values recorded for F5, and F10 were 0.9746 and 0.9644, respectively. This is consistent with a previous study [85], where apremilast (a drug approved for the treatment of psoriasis or psoriatic arthritis) encapsulated into PLGA NPs showed a bi-phasic drug release, with the second phase sustaining drug release up to 2 days, and its release kinetics was also best fitting Higuchi kinetics.

### 3.2. Antibacterial Study

#### 3.2.1. Susceptibility Test and Determination of MIC for Free AZI

The susceptibility of tested bacterial strains toward free AZI was determined following the disc diffusion method [67]. Obtained data revealed the resistance of *P. aeruginosa,* MRSA, and *E.* faecalis to AZI. MIC values were 256, 256, and >1000 µg/mL for *P. aeruginosa*, MRSA, and *E. faecalis*, respectively. Resistance of *P. aeruginosa* to free AZI may be traced back to low membrane permeability and active efflux of antibiotics [86]. *P. aeruginosa* was reported to have a lower outer membrane permeability than other Gram-negative bacteria [87,88], in addition to the relative dearth in porin channels which decreases the penetration of AZI across cell walls [86]. Modification of the drug target, inactivation of the drug, and active efflux might be possible resistance mechanisms to free AZI in Gram-positive bacteria. [89].

#### 3.2.2. Investigation of Bacterial Efflux Activity

##### Determination of Efflux Activity by Cartwheel Method

**Figure 4:**
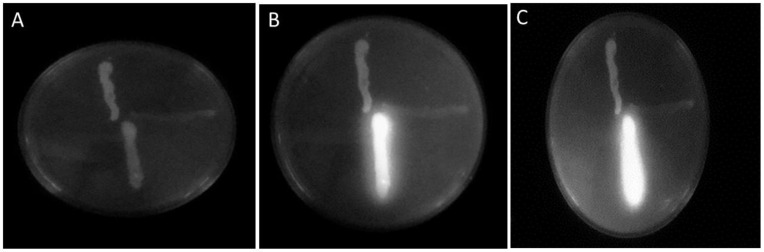
The ethidium bromide (EtBr) agar cartwheel method applied to tested bacterial strain, MRSA (up), *E. faecalis* (right), and *P. aeruginosa* (down) on agar plates contained different concentrations of EtBr (**A**) 0 µg/mL, (**B**) 1 µg/mL, and (**C**) 2 µg/mL. Only *P. aeruginosa* retained EtBr and was fluorescence after exposure to UV light due to a lack of the efflux mechanism contrary to the absence of fluorescence in both MRSA and *E. faecalis*, due to the presence of efflux mechanism in these bacterial strains resulting in complete efflux of EtBr.

Bacterial strains were screened for their efflux activity by investigating their ability to release different concentrations of EtBr. As presented in Figure 4, both *E. faecalis* and MRSA completely efflux EtBr out of cells when grown on TSA plates containing different concentrations of EtBr (up to 2 µg/mL). However, *P. aeruginosa* retained EtBr intracellularly when it grew on TSA plate containing EtBr concentration of 0.5 µg/ml, and the bacteria were fluorescing on the plate (see Video S1). Thus, the efflux mechanism was considered to be one of the resistant mechanisms developed by *E. faecalis* and MRSA against AZI. The obtained data are matched with a previous study [68], where *E. faecalis* and MRSA were reported to have efflux mechanisms and were reported to fluoresce at 1.5 and 2 µg/mL, respectively. The lack of efflux mechanism in *P. aeruginosa* is also consistent with a previous study, reported that 12 out of 25 strains of *P. aeruginosa* retained EtBr at a concentration lower than 0.5 µg/ml, indicating that efflux mechanism was detected only in 53.5% of tested *P. aeruginosa* strains [90].

##### Determination of AZI Efflux via Efflux Pump Inhibitor (EPI)

The MIC value of free AZI was also determined in the presence of VP, a known efflux pump inhibitor of prokaryotic efflux systems [71,91,92] as a further confirmatory test for the presence or absence of efflux pump in tested bacterial strain by using microdilution assay.

For *P. aeruginosa*, we found that the MIC value of free AZI did not change in the presence of VP and this confirmed the absence of an efflux mechanism. Contrary to a 32-fold (from 256 to 8 µg/mL) decrease of MIC values for free AZI was recorded in the presence of VP for both MRSA and *E. faecalis*, and thereby confirming the presence of an efflux mechanism in these bacteria. Our results are consistent with a previous study [93], where, VP was used as EPI, and demonstrated an improvement of antibacterial activity for AZI and rifampicin [91,94].

#### 3.2.3. Cytotoxicity Test

In the current study, the cytotoxicity test of AZI, F5, and F10 was performed on Wi-38 cells, human lung fibroblast cell lines, to investigate their safety. As presented in Table 2, the concentration responsible for the death of 50% of cells (CC50) was determined to assess the in vitro safety of free AZI versus nano-formulations. CC50 values for the free AZI, F5, and F10 were 1161.47 ± 270.71, 3138.59 ± 217.42, and 7304, 51 ± 5812.64 µg/mL, respectively. As revealed, the CC50 value for F5 non-significantly (*p* > 0.05) differed from CC50 for F10, however, both values are significantly (*p* < 0.05) differing from CC50 value for free AZI. This is indicative of the less in vitro cytotoxicity of nano-formulations compared to free AZI.

#### 3.2.4. Antibacterial Activity of AZI-PLGA NPs

To ascertain the efficacy of PNPs to bypass the efflux mechanism, the MIC values for free AZI and AZI-PLGA NPs (F5 and F10 formulations) were determined by microdilution assay, as described in Section 2.2.4. MIC values recorded for free AZI, F5 and F10 formulations with *P. aeruginosa*, MRSA and *E. faecalis* were presented in Table 2.

It is worth noting that bacterial strains treated with Blank PLGA NPs did not show any antibacterial activity, thus, any improvement of antibacterial activity observed with F5 and F10 formulations against tested bacteria could be attributed to encapsulated AZI and the ability of nano-formulation to skip the efflux mechanism present in MRSA and *E. faecalis*. As presented in Table 2, no significant differences (*p* > 0.05) were observed for the antibacterial activity of F5 and F10 on tested bacterial strains. This might be attributed to the presence of very few molecules of Tween 80 at the surface of F5 formulation that are unable to impart any changes on the surface properties of NPs to affect its interaction with the bacteria.

As presented in Table 2, MIC values recorded for free AZI, and nano-formulations were less than their CC50 values except for free AZI with *E. faecalis*. Gram-positive bacteria had a higher sensitivity towards NPs than Gram-negative bacteria as revealed by the four-fold decrease of MIC values recorded for Gram-positive bacteria when treated with NPs formulations. MIC significantly (*p* < 0.05) decreased from 256 to 64 µg/mL and from >1000 to 256 µg/mL in MRSA and *E. faecalis*, respectively, with no reduction of MIC value (256 µg/mL) observed in *P. aeruginosa*. Thus, AZI-PLGA NPs were able to overcome the bacterial efflux mechanism. Although MRSA and *E. faecalis* were demonstrated to have efflux mechanisms, it is worth noting that other resistant mechanisms might be present in these bacteria [57,95].

The improvement of antibacterial activity recorded with AZI-PLGA NPs matched the results reported previously by Aboutaleb and colleagues [48], where free rifampicin inhibited the growth of mycobacteria effectively at a concentration equivalent to 22 μg/mL, while Rifampicin loaded solid lipid nanoparticles inhibited the bacterial growth effectively at a concentration equivalent to 2.75 μg/mL. In other words, rifampicin-loaded solid lipid nanoparticles reduced the MIC values by eight times compared to the MIC value of free rifampicin. Mycobacteria was reported to resist antibiotics via two mechanisms and they are the cell wall permeability barrier and the active multidrug efflux pumps [96]. Our results were also consistent with a study performed by Moazeni and colleagues [47] where *Candida albicans*, *Candida parapsilosis*, and *Candida glabrata* were resistant to free fluconazole, and it was reported that MIC for free fluconazole was ≥64 µg/mL. However, by encapsulating fluconazole into solid lipid nanoparticles, MIC was reduced to be ≤8 µg/mL. Overexpression of plasma membrane transport proteins that pump the azoles out of cells is a frequent mechanism of high-level azole resistance in fungi, thereby reducing the intracellular concentration of azole in yeast, rendering it inactive [97].

The inactivity of AZI-PLGA NPs towards *P. aeruginosa* might be attributed to the nature of the cell wall. The cell wall of Gram-negative and Gram-positive bacteria is composed of a peptidoglycan (sugar/amino acid polymer) layer, however, it is thicker in Gram-negative bacteria than Gram-positive bacteria. The double membrane envelope of Gram-negative bacteria prevents many antibiotics from accessing their targets [58,59,98,99].

Additionally, the surface charge was reported to be one of the determinant factors for the antimicrobial activity of NPs [100]. The net negative charge of the cell wall of Gram-negative bacteria is more negatively charged than the cell wall of Gram-positive bacteria [101]. Thus, upon approaching AZI-PLGA NPs that were carrying a negative surface charge to the cell wall of Gram-negative bacteria, repulsion forces rather than attraction forces were dominating [102], and this might be responsible for hindering the adhesion of NPs onto the cell wall with *P. aeruginosa* and thus inhibited NPs’ entry into the bacteria [102]. Contrary to Gram-positive bacteria, attraction forces were dominating rather than repulsion forces, and this favored the adhesion of NPs onto the cell wall facilitating its entry into bacteria.

Many binding forces were reported to be involved in the NPs adhesion to the bacterial cell wall to facilitate its entry into bacteria and they are; electrostatic, van der Waals, and hydrogen bonding interactions [103]. Amphiphilic molecules embedded in the walls of Gram-negative (e.g., lipopolysaccharides and phospholipids) and Gram-positive (e.g., teichoic acid and lipoteichoic acid) bacteria are the first molecules involved in binding with NPs [104]. These amphiphilic molecules have a hydrophilic and a hydrophobic region that are able to interact with NPs approaching the cell wall depending on the material composition and the net surface charges of NPs [7]. These binding forces might be involved in NPs’ adhesion onto the cell wall of MRSA and *E. faecalis.*

The size of NPs is also a very crucial factor for antibacterial activity, although the impact of particle size on the antibacterial activity of metallic NPs has been explored [105,106,107], little is known about their correlation with polymeric NPs [108], and this demands further investigations. However, few studies have demonstrated that PNPs sized less than 200 nm showed enhanced membrane permeability, and thereby, superior antimicrobial activity compared to PNPs of a bigger size [34,108]. In the current study, AZI-PLGA NPs sized 134 to 107 nm for F5 and F10 formulations, respectively, reduced MIC by four-fold and this matched results reported in a previous study [34], where imipenem poly Ɛ-caprolactone (PCL) nanoparticles IMP/PCL sized 132 ± 20 nm had better antimicrobial activity against imipenem resistant isolates of *P. aeruginosa* and *K. pneumoniae* than imipenem PLGA NPs sized 348 ± 65. MIC values recorded for IMP/PCL and IMP/PLGA were five-fold and two-fold less than the MIC value of free IMP, respectively. The better antibacterial activity of IMP/PCL was attributed to the smaller size of IMP/PCL that facilitated the diffusion of NPs across the bacterial cell wall [34].

Therefore, for the purpose of bacteria eradication, the antimicrobial agent is required to be inside a microbial system for a longer period at high concentrations. Bacterial strains have efflux pumps that are able to expel the antibiotic, thus, leaving an inadequate amount of the antibiotic for proper antibacterial action and resulting in the failure of antimicrobial therapy. The current study demonstrated that encapsulation of antibiotics into PNPs could overcome the bacterial efflux mechanism and retain the antibiotic for a longer time inside the bacteria due to the ability of PLGA NPs to sustain drug release. However, further investigations are necessary to be performed in the future to explore the correlation (if any) of particle size, surface charge, and material composition of PNPs on their antimicrobial activity.

## 4. Conclusions

The crisis of antibiotic resistance is a worldwide public health concern that has a negative impact on healthcare and the economy. This demands the discovery of new antimicrobial agents or finding novel strategies to combat antimicrobial resistance. Discovery of new antimicrobial agents is a very expensive, long journey process with a high probability of developing bacterial resistance towards the newly developed antimicrobial agents. Nanoparticles are known to overcome efflux resistance mechanisms towards several chemotherapeutic agents. In this study, AZI was encapsulated into a biocompatible PLGA polymer by nano-precipitation method, several parameters including drug to polymer ratio, surfactant, and pH of aqueous medium were investigated to maximize drug loading and control particle size to be around 100 nm to maximize interaction with bacteria. Nano-formulations demonstrated to have a higher value of CC50 compared to free AZI and thus are safer than free AZI. A dramatic improvement of antibacterial activity was recorded for AZI-PLGA NPs over free AZI, where the MIC values of AZI-PLGA NPs were four times less than the MIC value of free AZI. MIC value reduced from >1000 to 256 µg/mL and from 256 to 64 µg/mL for *E. faecalis* and MRSA, respectively, contrary to no change detected for MIC values in the case of *P. aeruginosa*. Notably, *E. faecalis* and MRSA were found to possess the efflux mechanism, while it was absent in *P. aeruginosa*. These results revealed that AZI-PLGA NPs retained AZI antibacterial activity against resistant bacteria carrying efflux mechanism. This gave a new hope to manage infections in the future, however, further investigations are necessary to be performed in the future to explore the correlation (if any) of particle size, surface charge, and material composition of PNPs on their antimicrobial activity.

## Figures and Tables

**Table 1:** * Physicochemical characteristics of AZI PLGA NPs, each value is an average of three replicates.

AZI Initial Weight				
(mg)/Formula Name	Aqueous Phase	** %DL ± SD	** PS (D nm) ± SD (PDI)	** ZP (mV) ± SD
Blank	Water	------	188.5 ± 51.92 (0.193)	−24.83 ± 6.85
10/F1	Water	3.88 ± 0.07	225.5 ± 128.7 (0.276)	−27.9 ± 6.61
15/F2	Water	3.43 ± 0.11	184.5 ± 115 (0.253)	−30.1 ± 6.61
20/F3	Water	3.47 ± 0.24	101 ± 25.01 (0.181)	−34.8 ± 6.61
50/F4	Water	3.52 ± 0.23	593.7 ± 244.4 (0.651)	−29.5 ± 6.61
10/F5	Water/Tween 80 (0.1%)	5.74 ± 0.28	134 ± 57.53 (0.198)	−32.5.2 ± 4.87
15/F6	Water/Tween 80 (0.1%)	3.43 ± 0.14	114.3 ± 52.76 (0.266)	−35.7 ± 6.61
20/F7	Water/Tween 80 (0.1%)	4 ± 0.16	112.3 ± 42.08 (0.279)	−34.2 ± 6.61
50/F8	Water/Tween 80 (0.1%)	2.88 ± 0.34	154.9 ± 78.03 (0.269)	−30.2 ± 6.61
10/F9	Phosphate buffer (10 mM PH 6)	3.52 ± 0.23	118.4 ± 56.27 (0.204)	−32.7 ± 6.45
15/F10	Phosphate buffer (10 mM PH 6)	4.78 ± 0.13	107.5 ± 57.97 (0.307)	−33.3 ± 6.62
20/F11	Phosphate buffer (10 mM PH 6)	3.78 ± 0.28	105.8 ± 42.6 (0.367)	−30.9 ± 5.34
50/F12	Phosphate buffer (10 mM, PH 6)	3.35 ± 0.16	521.6 ± 158.4 (0.598)	−31.6 ± 6.3
10/F13	Phosphate buffer (10 mM PH7.4)	4.59 ± 0.27	111.4 ± 42.95 (0.301)	−41.8 ± 5.79
15/F14	Phosphate buffer (10 mM PH7.4)	4.35 ± 0.12	142.2 ± 58.73 (0.224)	−35.2.1 ± 5.87
20/F15	Phosphate buffer (10 mM PH7.4)	4.55 ± 0.26	154.3 ± 98.39 (0.316)	−34.7 ± 8.08
50/F16	Phosphate buffer (10 mM PH7.4)	4.32 ± 0.45	416 ± 50 (0.595)	−43.6 ± 6.65
10/F17	Phosphate buffer (10 mM PH 8)	3.19 ± 0.18	142.6 ± 67.46 (0.272)	−52.5 ± 7.8
15/F18	Phosphate buffer (10 mM PH 8)	3.49 ± 0.25	348.8 ± 104.1 (0.584)	−41.1 ± 4.99
20/F19	Phosphate buffer (10 mM PH 8)	4 ± 0.67	534.3 ± 246.7 (0.626)	−40.9 ± 5.06
50/F20	Phosphate buffer (10 mM PH 8)	3.13 ± 0.07	673 ± 151 (0.437)	−56.1 ± 8.06

* Data were analyzed by two-way ANOVA; there was non-significant (*p* > 0.05) differences between samples concerning all measured physicochemical parameters. ** Physicochemical parameters measured were; percentage of drug loading (DL%), particle size (PS), Diameter in nm (D nm), polydispersity index (PDI), zeta potential (ZP), standard deviation (SD).

**Table 2:** * MIC values of free AZI and AZI PLGA NPs (F5 and F10) recorded for *P. aeruginosa* MRSA, and *E. faecalis*, and their ** CC50 on Wi-38 cells, human lung fibroblast cell lines.

Tested Sample	*P. aeruginosa*	MRSA	*E. faecalis*
Free AZI	256	256	>1000
F5	256	64 ^a^	256 ^a^
F10	256	64 ^a^	256 ^a^

* MIC: minimum inhibitory concentration; ** CC50: cytotoxic concentrations of free AZI, F5, and F10 responsible for the death of 50% of Wi-38 cells is equivalent to 1161.47 ± 270.71, 3138.59 ± 217.42, and 7304, 51 ± 5812.64 (µg/mL), respectively. Data were analyzed by using one-way ANOVA. “^a^” means a significant (*p* < 0.05) differences between AZI-PLGA NPs when compared to free AZI.

## Data Availability

All authors are happy to share all data (including Supplementary Materials).

## Supplementary Materials

The following supporting information can be downloaded at: https://www.mdpi.com/article/10.3390/antibiotics11111623/s1, Video S1: Determination of Efflux Activity by Cartwheel Method.

## References

[1] Founou R.C., Founou L.L., Essack S.Y. (2017). Clinical and Economic Impact of Antibiotic Resistance in Developing Countries: A Systematic Review and Meta-Analysis. PLoS ONE.

[2] Dadgostar P. (2019). Antimicrobial Resistance: Implications and Costs. Infect. Drug Resist..

[3] Singh P., Garg A., Pandit S., Mokkapati V.R.S.S., Mijakovic I. (2018). Antimicrobial Effects of Biogenic Nanoparticles. Nanomaterials.

[4] Pelgrift R.Y., Friedman A.J. (2013). Nanotechnology as a Therapeutic Tool to Combat Microbial Resistance. Adv. Drug Deliv. Rev..

[5] Hutchings M.I., Truman A.W., Wilkinson B. (2020). ScienceDirect Antibiotics : Past, Present and Future. Curr. Opin. Microbiol..

[6] Upadhya R.K., Shenoy L., Venkateswaran R. (2018). Effect of Intravenous Dexmedetomidine Administered as Bolus or as Bolus-plus-Infusion on Subarachnoid Anesthesia with Hyperbaric Bupivacaine. J. Anaesthesiol. Clin. Pharmacol..

[7] Abo-zeid Y., Williams G.R. (2020). The Potential Anti-Infective Applications of Metal Oxide Nanoparticles: A Systematic Review. Wiley Interdiscip. Rev. Nanomed. Nanobiotechnol..

[8] Teixeira M.C., Sanchez-Lopez E., Espina M., Calpena A.C., Silva A.M., Veiga F.J., Garcia M.L., Souto E.B. (2018). Advances in Antibiotic Nanotherapy.

[9] Magiorakos A.P., Srinivasan A., Carey R.B., Carmeli Y., Falagas M.E., Giske C.G., Harbarth S., Hindler J.F., Kahlmeter G., Olsson-Liljequist B. (2012). Multidrug-Resistant, Extensively Drug-Resistant and Pandrug-Resistant Bacteria: An International Expert Proposal for Interim Standard Definitions for Acquired Resistance. Clin. Microbiol. Infect..

[10] Ebbensgaard A.E., Løbner-Olesen A., Frimodt-Møller J. (2020). The Role of Efflux Pumps in the Transition from Low-Level to Clinical Antibiotic Resistance. Antibiotics.

[11] Blecher K., Nasir A., Friedman A. (2011). The Growing Role of Nanotechnology in Combating Infectious Disease. Virulence.

[12] Lamut A., Peterlin Mašič L., Kikelj D., Tomašič T. (2019). Efflux Pump Inhibitors of Clinically Relevant Multidrug Resistant Bacteria. Med. Res. Rev..

[13] Annunziato G. (2019). Strategies to Overcome Antimicrobial Resistance (AMR) Making Use of Non-Essential Target Inhibitors: A Review. Int. J. Mol. Sci..

[14] Ritsema J.A.S., Van Der Weide H., Te Welscher Y.M., Goessens W.H.F., Van Nostrum C.F., Storm G., Bakker-Woudenberg I.A.J.M., Hays J.P. (2018). Antibiotic-Nanomedicines: Facing the Challenge of Effective Treatment of Antibiotic-Resistant Respiratory Tract Infections. Future Microbiol..

[15] Ho D.K., Nichols B.L.B., Edgar K.J., Murgia X., Loretz B., Lehr C.M. (2019). Challenges and Strategies in Drug Delivery Systems for Treatment of Pulmonary Infections. Eur. J. Pharm. Biopharm..

[16] Mondorf A.W., Breier J., Hendus J., Scherberich J.E., Mackenrodt G., Shah P.M., Stille W., Schoeppe W. (1979). The Effect of Aminoglycosides on Proximal Tubular Membranes of Human Kidneys. Infection.

[17] Prayle A., Watson A., Fortnum H., Smyth A. (2010). Side Effects of Aminoglycosides on the Kidney, Ear and Balance in Cystic Fibrosis. Thorax.

[18] Yu T., Jiang G., Gao R., Chen G., Ren Y., Liu J., van der Mei H.C., Busscher H.J. (2020). Circumventing Antimicrobial-Resistance and Preventing Its Development in Novel, Bacterial Infection-Control Strategies. Expert Opin. Drug Deliv..

[19] Abo-zeid Y., Bakkar M.R., Elkhouly G.E., Raya N.R., Zaafar D. (2022). Rhamnolipid Nano-Micelles Versus Alcohol-Based Hand Sanitizer : A Comparative Study for Antibacterial Activity against Hospital-Acquired Infections and Toxicity Concerns. Antibiotics.

[20] Sobhy Y., Mady M., Mina S., Abo-zeid Y. (2022). Phytochemical and Pharmacological Values of Two Major Constituents of Asparagus Species and Their Nano Formulations: A Review. J. Adv. Pharm. Res..

[21] Abo-zeid Y., Williams G.R., Touabi L., Mclean G.R. (2020). An Investigation of Rhinovirus Infection on Cellular Uptake of Poly (Glycerol-Adipate) Nanoparticles. Int. J. Pharm..

[22] Abo-zeid Y., Diab R., Sanad R., Skran W. (2021). Recent Advances in Herbal-Based Nanomedicine for Anti-Inflammatory Purposes. J. Adv. Pharm. Res..

[23] Hashim F., El-Ridy M., Nasr M., Abdallah Y. (2010). Preparation and Characterization of Niosomes Containing Ribavirin for Liver Targeting. Drug Deliv..

[24] Ali A.M., Hill H.J., Elkhouly G.E., Bakkar M.R., Raya N.R., Stamataki Z., Abo-zeid Y. (2022). Rhamnolipid Nano-Micelles Inhibit SARS-CoV-2 Infection and Have No Dermal or Eye Toxic Effects in Rabbits. Antibiotics.

[25] Abo-zeid Y., Urbanowicz R.A., Thomson B.J., William L., Tarr A.W., Garnett M.C. (2018). Enhanced Nanoparticle Uptake into Virus Infected Cells: Could Nanoparticles Be Useful in Antiviral Therapy?. Int. J. Pharm..

[26] Abo-zeid Y., Mantovani G., Irving W.L., Garnett M.C. (2018). Synthesis of Nucleoside-Boronic Esters Hydrophobic pro-Drugs: A Possible Route to Improve Hydrophilic Nucleoside Drug Loading into Polymer Nanoparticles. J. Drug Deliv. Sci. Technol..

[27] Abo-Zeid Y., Ismail N.S., McLean G.R., Hamdy N.M. (2020). A Molecular Docking Study Repurposes FDA Approved Iron Oxide Nanoparticles to Treat and Control COVID-19 Infection. Eur. J. Pharm. Sci..

[28] Mba I.E., Nweze E.I. (2021). Nanoparticles as Therapeutic Options for Treating Multidrug-Resistant Bacteria: Research Progress, Challenges, and Prospects. World J. Microbiol. Biotechnol..

[29] Kirtane A.R., Verma M., Karandikar P., Furin J., Langer R., Traverso G. (2021). Nanotechnology Approaches for Global Infectious Diseases. Nat. Nanotechnol..

[30] Bakkar M.R., Faraag A.H.I., Soliman E.R.S., Fouda M.S., Sarguos A.M.M., McLean G.R., Hebishy A.M.S., Elkhouly G.E., Raya N.R., Abo-zeid Y. (2021). Rhamnolipids Nano-Micelles as a Potential Hand Sanitizer. Antibiotics.

[31] Abo-zeid Y., Amer A., El-Houssieny B., Bakkar M., Sakran W. (2021). Overview on Bacterial Resistance and Nanoparticles to Overcome Bacterial Resistance. J. Adv. Pharm. Res..

[32] Garnett M.C., Kallinteri P. (2006). Nanomedicines and Nanotoxicology: Some Physiological Principles. Occup. Med..

[33] Burgess K., Li H., Abo-Zeid Y., Fatimah, Williams G.R. (2018). The Effect of Molecular Properties on Active Ingredient Release from Electrospun Eudragit Fibers. Pharmaceutics.

[34] Shaaban M.I., Shaker M.A., Mady F.M. (2017). Imipenem/Cilastatin Encapsulated Polymeric Nanoparticles for Destroying Carbapenem-Resistant Bacterial Isolates. J. Nanobiotechnol..

[35] Derbali R.M., Aoun V., Moussa G., Frei G., Tehrani S.F., Del’Orto J.C., Hildgen P., Roullin V.G., Chain J.L. (2019). Tailored Nanocarriers for the Pulmonary Delivery of Levofloxacin against Pseudomonas Aeruginosa: A Comparative Study. Mol. Pharm..

[36] Baptista P.V., McCusker M.P., Carvalho A., Ferreira D.A., Mohan N.M., Martins M., Fernandes A.R. (2018). Nano-Strategies to Fight Multidrug Resistant Bacteria—“A Battle of the Titans”. Front. Microbiol..

[37] Abo-zeid Y., Garnett M.C. (2020). Polymer Nanoparticle as a Delivery System for Ribavirin: Do Nanoparticle Avoid Uptake by Red Blood Cells?. J. Drug Deliv. Sci. Technol..

[38] Huh A.J., Kwon Y.J. (2011). “Nanoantibiotics”: A New Paradigm for Treating Infectious Diseases Using Nanomaterials in the Antibiotics Resistant Era. J. Control. Release.

[39] Zhao J., Stenzel M.H. (2018). Entry of Nanoparticles into Cells: The Importance of Nanoparticle Properties. Polymer Chemistry.

[40] Behzadi S., Serpooshan V., Tao W., Hamaly M.A., Mahmoud Y., Dreaden E.C., Brown D., Alkilany A.M., Omid C., Mahmoudi M. (2018). HHS Public Access. Cellular uptake of nanoparticles: Journey inside the cell. Chem. Soc. Rev..

[41] Eleraky N.E., Allam A., Hassan S.B., Omar M.M. (2020). Nanomedicine Fight against Antibacterial Resistance: An Overview of the Recent Pharmaceutical Innovations. Pharmaceutics.

[42] Hasani A., Madhi M., Gholizadeh P., Shahbazi Mojarrad J., Ahangarzadeh Rezaee M., Zarrini G., Samadi Kafil H. (2019). Metal Nanoparticles and Consequences on Multi-Drug Resistant Bacteria: Reviving Their Role. SN Appl. Sci..

[43] Sun M., Zhu C., Long J., Lu C., Pan X., Wu C. (2020). PLGA Microsphere-Based Composite Hydrogel for Dual Delivery of Ciprofloxacin and Ginsenoside Rh2 to Treat Staphylococcus Aureus-Induced Skin Infections. Drug Deliv..

[44] Gupta D., Singh A., Khan A.U. (2017). Nanoparticles as Efflux Pump and Biofilm Inhibitor to Rejuvenate Bactericidal Effect of Conventional Antibiotics. Nanoscale Res. Lett..

[45] Salas Orozco M.F., Niño-Martínez N., Martínez-Castañón G.A., Méndez F.T., Ruiz F. (2019). Molecular Mechanisms of Bacterial Resistance to Metal and Metal Oxide Nanoparticles. Int. J. Mol. Sci..

[46] Raza A., Kamato D., Sime F.B., Roberts J.A., Popat A., Falconer J.R., Kumeria T. (2022). Influence of PEGylated Porous Silicon Nanoparticles on Permeation and Efflux of an Orally Administered Antibiotic. Mater. Today Adv..

[47] Moazeni M., Kelidari H.R., Saeedi M., Morteza-Semnani K., Nabili M., Gohar A.A., Akbari J., Lotfali E., Nokhodchi A. (2016). Time to Overcome Fluconazole Resistant Candida Isolates: Solid Lipid Nanoparticles as a Novel Antifungal Drug Delivery System. Colloids Surf. B Biointerfaces.

[48] Aboutaleb E., Noori M., Gandomi N., Atyabi F., Fazeli M.R., Jamalifar H., Dinarvand R. (2012). Improved Antimycobacterial Activity of Rifampin Using Solid Lipid Nanoparticles. Int. Nano Lett..

[49] Spirescu V.A., Chircov C., Grumezescu A.M., Andronescu E. (2021). Polymeric Nanoparticles for Antimicrobial Therapies: An up-to-Date Overview. Polymers.

[50] Murakami M., Cabral H., Matsumoto Y., Wu S., Kano M.R., Yamori T., Nishiyama N., Kataoka K. (2011). Improving Drug Potency and Efficacy by Nanocarrier-Mediated Subcellular Targeting. Sci. Transl. Med..

[51] Yao Y., Zhou Y., Liu L., Xu Y., Chen Q., Wang Y., Wu S., Deng Y., Zhang J., Shao A. (2020). Nanoparticle-Based Drug Delivery in Cancer Therapy and Its Role in Overcoming Drug Resistance. Front. Mol. Biosci..

[52] Hooda Y., Tanmoy A.M., Sajib M.S.I., Saha S. (2020). Mass Azithromycin Administration: Considerations in an Increasingly Resistant World. BMJ Glob. Health.

[53] Echeverría-Esnal D., Martin-Ontiyuelo C., Navarrete-Rouco M.E., De-Antonio Cuscó M., Ferrández O., Horcajada J.P., Grau S. (2021). Azithromycin in the Treatment of COVID-19: A Review. Expert Rev. Anti. Infect. Ther..

[54] Parnham M.J., Haber V.E., Giamarellos-Bourboulis E.J., Perletti G., Verleden G.M., Vos R. (2014). Azithromycin: Mechanisms of Action and Their Relevance for Clinical Applications. Pharmacol. Ther..

[55] Porco T.C., Gebre T., Ayele B., House J., Keenan J., Zhou Z., Hong K.C., Stoller N., Ray K.J., Emerson P. (2009). Effect of Mass Distribution of Azithromycin for Trachoma Control on Overall Mortality in Ethiopian Children: A Randomized Trial. JAMA J. Am. Med. Assoc..

[56] Doan T., Worden L., Hinterwirth A., Arzika A.M., Maliki R., Abdou A., Zhong L., Chen C., Cook C., Lebas E. (2020). Macrolide and Nonmacrolide Resistance with Mass Azithromycin Distribution. N. Engl. J. Med..

[57] Leclercq R. (2002). Mechanisms of Resistance to Macrolides and Lincosamides: Nature of the Resistance Elements and Their Clinical Implications. Clin. Infect. Dis..

[58] Vaara M. (1993). Outer Membrane Permeability Barrier to Azithromycin, Clarithromycin, and Roxithromycin in Gram-Negative Enteric Bacteria. Antimicrob. Agents Chemother..

[59] Schweizer H.P. (2003). Efflux as a Mechanism of Resistance to Antimicrobials in Pseudomonas Aeruginosa and Related Bacteria: Unanswered Questions. Genet. Mol. Res..

[60] Chevalier M.T., Gonzalez J., Alvarez V. (2015). Biodegradable Polymeric Microparticles as Drug Delivery Devices. IFMBE Proc..

[61] Xiong M.H., Bao Y., Yang X.Z., Zhu Y.H., Wang J. (2014). Delivery of Antibiotics with Polymeric Particles. Adv. Drug Deliv. Rev..

[62] Nader A., El-Hosseiny G., Elleboudy N., Yassein M. Prevalence and Antimicrobial Susceptibility Pattern of Enterococcus Sp. Isolated from Different Clinical Specimens from Some Hospitalized Patients in Egypt. Proceedings of the 8th Annual International Ain Shams University Congress.

[63] Mohammadi G., Valizadeh H., Barzegar-Jalali M., Lotfipour F., Adibkia K., Milani M., Azhdarzadeh M., Kiafar F., Nokhodchi A. (2010). Development of Azithromycin-PLGA Nanoparticles: Physicochemical Characterization and Antibacterial Effect against Salmonella Typhi. Colloids Surf. B Biointerfaces.

[64] Ghari T., Kobarfard F., Mortazavi S.A. (2013). Development of a Simple RP-HPLC-UV Method for Determination of Azithromycin in Bulk and Pharmaceutical Dosage Forms as an Alternative to the USP Method. Iran. J. Pharm. Res..

[65] Kakkar V., Kaur I.P., Kaur A.P., Saini K., Singh K.K. (2018). Topical Delivery of Tetrahydrocurcumin Lipid Nanoparticles Effectively Inhibits Skin Inflammation: In Vitro and in Vivo Study. Drug Dev. Ind. Pharm..

[66] Zhang Y., Huo M., Zhou J., Zou A., Li W., Yao C., Xie S. (2010). DDSolver : An Add-In Program for Modeling and Comparison of Drug Dissolution Profiles. AAPS J..

[67] CLSI (2016). CLSI Clinical and Laboratory Standards Institute: Performance Standards for Antimicrobial Susceptibility Testing Supplement M100S.

[68] Martins M., Viveiros M., Couto I., Costa S.S., Pacheco T., Fanning S., Pagès J.M., Amaral L. (2011). Identification of Efflux Pump-Mediated Multidrug-Resistant Bacteria by the Ethidium Bromide-Agar Cartwheel Method. In Vivo.

[69] Sharma A., Gupta V.K., Pathania R. (2019). Efflux Pump Inhibitors for Bacterial Pathogens: From Bench to Bedside. Indian J. Med. Res..

[70] Christena L.R., Mangalagowri V., Pradheeba P., Ahmed K.B.A., Shalini B.I.S., Vidyalakshmi M., Anbazhagan V., Subramanian N.S. (2015). Copper Nanoparticles as an Efflux Pump Inhibitor to Tackle Drug Resistant Bacteria. RSC Adv..

[71] Laws M., Shaaban A., Rahman K.M. (2019). Antibiotic Resistance Breakers: Current Approaches and Future Directions. FEMS Microbiol. Rev..

[72] Laudy A.E., Kulińska E., Tyski S. (2017). The Impact of Efflux Pump Inhibitors on the Activity of Selected Non-Antibiotic Medicinal Products against Gram-Negative Bacteria. Molecules.

[73] Putri D.C.A., Dwiastuti R., Marchaban M., Nugroho A.K. (2017). Optimization of Mixing Temperature and Sonication Duration in Liposomes Preparation. J. Pharm. Sci. Community.

[74] Piazzini V., D’Ambrosio M., Luceri C., Cinci L., Landucci E., Bilia A.R., Bergonzi M.C. (2019). Formulation of Nanomicelles to Improve the Solubility and the Oral Absorption of Silymarin. Molecules.

[75] Das P., Yang X.P., Ma L.Z. (2014). Analysis of Biosurfactants from Industrially Viable Pseudomonas Strain Isolated from Crude Oil Suggests How Rhamnolipids Congeners Affect Emulsification Property and Antimicrobial Activity. Front. Microbiol..

[76] Aucamp M., Odendaal R., Liebenberg W., Hamman J., Odendaal R., Liebenberg W., Hamman J. (2014). Amorphous Azithromycin with Improved Aqueous Solubility and Intestinal Membrane Permeability. Drug Dev. Ind. Pharm..

[77] Joshi A.S., Gahane A., Thakur A.K. (2016). Deciphering the Mechanism and Structural Features of Polysorbate 80 during Adsorption on PLGA Nanoparticles by Attenuated Total Re Fl Ectance—Fourier Transform Infrared Spectroscopy †. R. Soc. Chem..

[78] Graca M., Bongaerts J.H.H., Stokes J.R., Granick S. (2007). Friction and Adsorption of Aqueous Polyoxyethylene (Tween) Surfactants at Hydrophobic Surfaces. J. Colloid Interface Sci..

[79] Kallinteri P., Higgins S., Hutcheon G.A., St. Pourçain C.B., Garnett M.C. (2005). Novel Functionalized Biodegradable Polymers for Nanoparticle Drug Delivery Systems. Biomacromolecules.

[80] Sharma N., Madan P., Lin S. (2016). Effect of Process and Formulation Variables on the Preparation of Parenteral Paclitaxel-Loaded Biodegradable Polymeric Nanoparticles : A Co-Surfactant Study. Asian J. Pharm. Sci..

[81] Horwitz W. (2002). AOAC Guidelines for Single Laboratory Validation of Chemical Methods for Dietary Supplements and Botanicals.

[82] European Association for The Study of The Liver (2016). EASL Recommendations on Treatment of Hepatitis C. J. Hepatol..

[83] Fredenberg S., Wahlgren M., Reslow M., Axelsson A. (2011). The Mechanisms of Drug Release in Poly (Lactic-Co-Glycolic Acid)-Based Drug Delivery Systems—A Review. Int. J. Pharm..

[84] Mu L., Feng S. (2003). PLGA / TPGS Nanoparticles for Controlled Release of Paclitaxel : Effects of the Emulsifier and Drug Loading Ratio. Pharma. Res..

[85] Anwer M.K., Mohammad M., Ezzeldin E., Fatima F., Alalaiwe A., Iqbal M. (2019). Preparation of Sustained Release Apremilast-Loaded PLGAlga Nanoparticles: In Vitro Characterization and in Vivo Pharmacokinetic Study in Rats. Int. J. Nanomed..

[86] Langendonk R.F., Neill D.R., Fothergill J.L. (2021). The Building Blocks of Antimicrobial Resistance in Pseudomonas Aeruginosa: Implications for Current Resistance-Breaking Therapies. Front. Cell. Infect. Microbiol..

[87] Lister P.D., Wolter D.J., Hanson N.D. (2009). Antibacterial-Resistant Pseudomonas Aeruginosa: Clinical Impact and Complex Regulation of Chromosomally Encoded Resistance Mechanisms. Clin. Microbiol. Rev..

[88] Slama T.G. (2008). Gram-Negative Antibiotic Resistance: There Is a Price to Pay. Crit. Care.

[89] Portillo A., Ruiz-Larrea F., Zarazaga M., Alonso A., Martinez J.L., Torres C. (2000). Macrolide Resistance Genes in *Enterococcus* spp.. Antimicrob. Agents Chemother..

[90] Suresh M., Nithya N., Jayasree P.R., Manish Kumar P.R. (2016). Detection and Prevalence of Efflux Pump-Mediated Drug Resistance in Clinical Isolates of Multidrug-Resistant Gram-Negative Bacteria from North Kerala, India. Asian J. Pharm. Clin. Res..

[91] Seral C., Carryn S., Tulkens P.M., Van Bambeke F. (2003). Influence of P-Glycoprotein and MRP Effux Pump Inhibitors on the Intracellular Activity of Azithromycin and Ciprofloxacin in Macrophages Infected by Listeria Monocytogenes or Staphylococcus Aureus. J. Antimicrob. Chemother..

[92] Mullin S., Mani N., Grossman T.H. (2004). Inhibition of Antibiotic Efflux in Bacteria by the Novel Multidrug Resistance Inhibitors Biricodar (VX-710) and Timcodar (VX-853). Antimicrob. Agents Chemother..

[93] Pule C.M., Sampson S.L., Warren R.M., Black P.A., van Helden P.D., Victor T.C., Louw G.E. (2016). Efflux Pump Inhibitors: Targeting Mycobacterial Efflux Systems to Enhance TB Therapy. J. Antimicrob. Chemother..

[94] De Oliveira Demitto F., Do Amaral R.C.R., Maltempe F.G., Siqueira V.L.D., De Lima Scodro R.B., Lopes M.A., Caleffi-Ferracioli K.R., Canezin P.H., Cardoso R.F. (2015). In Vitro Activity of Rifampicin and Verapamil Combination in Multidrug-Resistant Mycobacterium Tuberculosis. PLoS ONE.

[95] Zhang Q., Plummer P.J. (2014). Mechanisms of Antibiotic Resistance in Campylobacter.

[96] De Rossi E., Aínsa J.A., Riccardi G. (2006). Role of Mycobacterial Efflux Transporters in Drug Resistance: An Unresolved Question. FEMS Microbiol. Rev..

[97] Das S., Ng W.K., Tan R.B.H. (2012). Are Nanostructured Lipid Carriers (NLCs) Better than Solid Lipid Nanoparticles (SLNs): Development, Characterizations and Comparative Evaluations of Clotrimazole-Loaded SLNs and NLCs?. Eur. J. Pharm. Sci..

[98] Gao M., Long X., Du J., Teng M., Zhang W., Wang Y., Wang X., Wang Z., Zhang P., Li J. (2020). Enhanced Curcumin Solubility and Antibacterial Activity by Encapsulation in PLGA Oily Core Nanocapsules. Food Funct..

[99] Cabeen M.T., Jacobs-Wagner C. (2005). Bacterial Cell Shape. Nat. Rev. Microbiol..

[100] Raghunath A., Perumal E. (2017). Metal Oxide Nanoparticles as Antimicrobial Agents: A Promise for the Future. Int. J. Antimicrob. Agents.

[101] Beveridge T.J. (1999). Sructure of Fram-Negative Cell Walls and Their Derived Mebrane Vesicles. J. Bacteriol..

[102] Dakal T.C., Kumar A., Majumdar R.S., Yadav V. (2016). Mechanistic Basis of Antimicrobial Actions of Silver Nanoparticles. Front. Microbiol..

[103] Parikh S.J., Chorover J. (2006). ATR-FTIR Spectroscopy Reveals Bond Formation During Bacterial Adhesion to Iron Oxide. Langmuir.

[104] Makin S.A., Beveridge T.J. (1996). The Influence of A-Band and B-Band Lipopolysaccharide on the Surface Characteristics and Adhesion of Pseudomonas Aeruginosa to Surfaces. Microbiology.

[105] Wu M., Guo H., Liu L., Liu Y., Xie L. (2019). Size-Dependent Cellular Uptake and Localization Profiles of Silver Nanoparticles. Int. J. Nanomed..

[106] Applerot G., Lellouche J., Lipovsky A., Nitzan Y., Lubart R., Gedanken A., Banin E. (2012). Understanding the Antibacterial Mechanism of CuO Nanoparticles: Revealing the Route of Induced Oxidative Stress. Small.

[107] Pareek V., Gupta R., Panwar J. (2018). Do Physico-Chemical Properties of Silver Nanoparticles Decide Their Interaction with Biological Media and Bactericidal Action? A Review. Mater. Sci. Eng. C.

[108] Hwang J., Mros S., Gamble A.B., Tyndall J.D.A., McDowell A. (2022). Improving Antibacterial Activity of a HtrA Protease Inhibitor JO146 against Helicobacter Pylori: A Novel Approach Using Microfluidics-Engineered PLGA Nanoparticles. Pharmaceutics.

